# Social ties and embeddedness in old age: older Turkish labour migrants in Vienna

**DOI:** 10.1080/1369183X.2016.1238907

**Published:** 2016-11-01

**Authors:** Monika Palmberger

**Affiliations:** ^a^ Department of Social and Cultural Anthropology, University of Vienna, Vienna, Austria

**Keywords:** Ageing, Turkish migrants, transnational ageing, social ties, social embeddedness, older migrants

## Abstract

This paper focuses on older Turkish labour migrants and their spouses, who mostly came to Vienna as young adults in the 1960s and thereafter. They are now entering retirement age and constitute a significant part of Vienna’s older population. I analyse their understandings of transnational ageing, their social ties and feelings of social embeddedness. For those still mobile, active participation in one of Vienna’s Turkish cultural/religious/political associations is identified as a particular source of social embeddedness. I argue that these voluntary associations provide an important place for older migrants to strengthen social ties and are relatively easy to access, including in old age. Nevertheless, I demonstrate that older Turkish labour migrants are exposed to several forms of discrimination, some of which are felt especially strongly in old age, including a lack of adequate institutionalised late life care. In the discussion of the paper, I critically revisit the debate on ethnicity as a resource versus ethnicity as a vulnerability factor in old age. I argue that this debate is misleading since it camouflages other central social categories and relations. I conclude by suggesting closer attention be paid to the specific but multiple generational experiences of older labour migrants and their spouses.

## Introduction

Austria, which has long been ignored the fact that it has become a country of immigration, has not yet fully acknowledged the diversity among its senior population. While in the past diversity in Austria’s capital city, Vienna, was less salient and primarily restricted to younger sections of the population, diversification among older people has now become apparent. With former ‘guest workers’ growing old, the demographic picture has changed, particularly in Vienna, where the majority of labour migrants who came to Austria (from Turkey and Yugoslavia) in the 1960s and thereafter have settled.^1^

Not only policy-makers lag behind with regard to these social realities. Until recently, social scientists have not met this challenge either: ‘Both “population ageing” and “international migration” have been studied intensively but rarely have the interactions between the two socio-demographic processes been examined’ (Warnes et al. 2004, 311).^2^ Even though there has been a steadily increasing interest in questions of ageing and migration, still only a few studies exist on this topic concerning Austria (see Reinprecht 2006). Migrant associations, which prove to be vital in the lives of older labour migrants and which therefore are central to the study presented here, are another under-researched subject in migration studies, particularly in Europe (Caglar 2006).

In this paper, I investigate the (transnational) understandings and ideas of ageing among Turkish labour migrants (and their spouses) and the ways they feel socially embedded. Contrary to literature highlighting the isolation older migrants face (see, e.g. Treas and Mazumdar 2002; Victor et al. 2001), I show that older migrants who have access to cultural, political and religious voluntary associations feel socially well-embedded. Nonetheless, I also show how older labour migrants are exposed to several forms of discrimination, some of which are felt especially strongly in old age.

While much points to the fact that older migrants are likely to face cumulative vulnerabilities (see, e.g. Baykara-Krumme, Schimany, and Motel-Klingebiel 2012; Dwyer and Papadimitriou 2006; Fennell, Phillipson, and Evers 1988; Jamieson 2002; Warnes, Warren, and Nolan 2000; White 2006), concentrating only on vulnerabilities prevents us from drawing a more differentiated picture that does justice to the diverse migrant groups with their different migration histories, present experiences and outlooks (see Vertovec 2007). Moreover, the sole focus on vulnerabilities, as inherent in the double jeopardy theory (see Dowd and Bengtson 1978; Ferraro and Farmer 1996), carries the risk that in the wider society older migrants are perceived first and foremost as a ‘social problem’. Torres (2006), for example, shows in the case of Sweden how older migrants are constructed as a ‘social problem’, as a homogeneous group that presents a major obstacle to care providers. This negative view of old age, however, has affected not only migrants but also non-migrants, since the older population in general has first and foremost been discussed in terms of its needs and deficits. This discussion has been supported by dominant gerontological theories, such as disengagement and activity theories, which respectively have described ageing as a loss of self-esteem and a withdrawal from society (see Fennell, Phillipson, and Evers 1988; Jamieson 2002).

Anthropologists have challenged the assumption that old age is universally a time of mutual withdrawal by old people from their surrounding societies (Keith 1980, 343; see also Cohen 1994; Pickard 1995; Sokolovsky 2009) and criticisms have been raised against such a pessimistic and problem-focused position on ageing and on older migrants in particular. By raising this challenge, scholars have drawn attention to the fact that migrants, even if they are at risk of multiple jeopardies, are agents who can draw on specific ‘ethnic’ resources that have been widely overlooked. In this way they have countered views on older migrants simply as victims of structural insecurities, and instead have stressed the active part older migrants take in creatively organising their lives. Scholars putting forward the ‘ethnicity as a resource’ argument stress that ethnicity presents itself as a special kind of wealth in the form of resources that enable the elderly to better manage their everyday lives and which can be drawn upon in times of crisis (Simić 1985, 67). Even when the focus on ‘ethnicity as a resource’ takes the life course into consideration, the debate still posits ethnicity as the ‘trigger’.

The idea of ethnicity as a resource versus a vulnerability factor is still implicit in many current (gerontological) works on older migrants, even when scholars do not always explicitly position themselves within this debate. By using ethnicity as a focus of analysis and by directly connecting ‘ethnicity’ (or what is seen as ethnicity) to positive or negative ‘outcomes’, scholars implicitly support an understanding of ethnic groups as homogeneous rather than as constructed and diverse (see Brubaker 2004). In this vein, ethnicity is too easily ascribed to migrants but not (or not to the same degree) to the main population in the host society. Such a biased approach has been critically investigated in discussions on ‘whiteness’, showing that ‘to leave whiteness unexamined is to perpetuate a kind of asymmetry that has marred even many critical analyses of racial formation and cultural practice’ (Frankenberg 1997, 1; see also Gilleard and Higgs 2014). This criticism also holds for the debate on Europe’s migrants where the host society is often presented as homogeneous and unmarked by history or practice.

In this paper, I assess social embeddedness in the largely understudied voluntary associations of which older labour migrants are active members. Rather than presenting migrant associations as ‘ethnic’, I draw attention to their diversity, particularly in terms of religion and political views. Here, social embeddedness is understood in a relational sense, as social relations that foster a sense of ‘rootedness’ and belonging, not in a geographically binding way but more in the form of social ties (see Brown 2002; Korinek, Entwisle, and Jampaklay 2005). Such an understanding of social embeddedness takes account of transnational ties and does not restrict a sense of embeddedness to a geographically defined place (e.g. Austria versus Turkey). Social ties important for my interlocutors’ sense of social embeddedness were closely linked to their everyday lives and daily routines, which included regular visits to one of the voluntary associations. Everyday life may be characterised as ‘what happens every day, the routine, repetitive taken-for-granted experiences, beliefs and practices; the mundane ordinary world, untouched by great events and the extraordinary’ (Featherstone 1992, 160–161). Everyday life is practised, rather than reflected upon, and this becomes most apparent when asking people about it. Crucial for understanding my interlocutors’ everyday lives was the fact that they did not divide their activities into daily routines and leisure time. When asked about what they did for recreation, they frankly referred back to their daily routines. While daily routines differed between men and women, for many of both groups these routines included visits to one of the Turkish associations or meeting other members of one of the associations. These routines took centre stage in the lives of the older migrants, who sincerely hoped to keep them up for as long as they were able.

## Methods and research contextualisation

The first guest worker contracts between Austria and Turkey were signed in 1964. Austria recruited mostly unskilled workers for the construction industry but also for other industries, including textile and paper industries, as well as for the tourism business (see Fassmann and Lichtenberger 1987). Their spouses (and children) often followed at a somewhat later stage. Even if there was a significant number of Turkish women who entered Austria as guest workers, the majority of my female interlocutors came to Austria in the context of family reunification. Some of the female migrants I interviewed had worked in the cleaning and catering industry after their arrival, two women had worked in home care but the majority had not entered the labour market at all. Most of my informants had only a basic education.^3^

For this study, labour migrants of 50 years and older are considered.^4^ Age, however, was less important than stage of life. My informants had either retired or were in the process of retiring (several of them applied for early retirement because of physical injuries). Entering retirement ended a phase of hard work as labour migrants and often coincided with their children moving out. This stage of life was also unique in the sense that it opened up new possibilities, especially for transnational living arrangements. Retirement was an important time when spouses renegotiated where they wished to live and how they wanted to divide their time between Turkey and Austria. These individual preferences regarding mobility were central to my informants’ understandings of a satisfactory old age but were also significantly restricted by existing mobility regimes (Glick Schiller and Salazar 2013). Especially when their retirement benefits were below the guaranteed minimum they risked losing compensation payments if they spent more than two months a year outside Austria.

When both men and women opted for some form of commuting between the two countries, there were some gender differences. While almost all my female informants wished to spend more time in Austria than in Turkey, because of their children and grandchildren, some of my male informants, although still a minority, were ready to spend the majority of their time in Turkey with regular visits to Austria. Despite gender differences, most couples in the end opted for one extended stay in Turkey, from one to four months (preferably during/including summertime) and spending the rest of the year with their children (and grandchildren) in Vienna. These findings correspond with those of other authors who have made similar observations in regard to gender differences (see, e.g. Bolzman, Kaeser, and Christe 2016).

The research presented in this paper is based on several complementary qualitative research methods, including participant observation, semi-structured narrative interviews (25), expert interviews (11) and informal interviews (30) conducted between 2013 and 2016. The semi-structured narrative interviews with Turkish migrants lasted between 45 minutes and 2 hours. With my key informants I conducted a second interview round. In these semi-structured interviews, I asked questions that invited interviewees to examine their present life by connecting it to past traits and future aspirations. The interviews thus give insight into past decisions, present life strategies and concerns as well as into hopes and fears regarding the future. The gender division of the 25 informants with whom I conducted in-depth interviews was almost equal, with 12 men and 13 women. All interviews were recorded and transcribed for reasons of storage and analysis.

Among my interviewees were practising Sunni Muslims, Alevi Muslims as well as atheists. While their family contexts varied, they were all married (only one of my informants was widowed) and had children, and in many cases grandchildren. Children, who generally had moved out of their parents’ home, did not live in the same building and sometimes not even in the same part of the city. Just over half of the people I talked to had Austrian citizenship; among this group there were more men than women. For those not yet awarded Austrian citizenship, the bureaucratic procedures and particularly the required German-language test constituted a hurdle too big to overcome. While language skills varied, men tended to speak German better than their wives. This is connected to the fact that men were more fully integrated into working life, and thus were confronted more often with the German language than women. Still, in many cases, language skills were basic and therefore the great majority of the narrative interviews were conducted in Turkish. Many of my interlocutors, particularly women, told me how much they regretted not having had the chance to learn German properly. They said that if they had the chance to come to Austria again, they would focus their energy on learning German and some of those who had not worked added that they would seek work if they had a second chance.

When the idea of recruiting guest workers for a limited period was first put forward in the early 1960s, no plans for integration measures such as language courses were included. The establishment of migrant associations, however, was supported by the authorities in the belief that these associations would be temporary and help guest workers to socialise in their new environment. However, in the 2000s such associations are still present throughout Vienna, and in 2004 there were 728 migrant associations registered in the city, 109 of which were organised by Turkish migrants (Sohler 2007, 379).

Besides the above-mentioned language difficulties and the resulting limitations on access to information, vulnerabilities my informants faced were first and foremost connected to health effects resulting from hard work, inadequate (and overpriced) housing, insecure working contracts and low-income/retirement benefits. The majority experienced difficulties in the transition from working life to retirement and either had to enter invalidity retirement, take early retirement or they faced long-term unemployment or very unstable employment (due to having worked via temporary employment agencies) in the years before retirement.

Murat,^5^ a man in his late fifties, exemplifies this difficult situation at the end of working life very well. Murat lives with his wife; they have three children and nine grandchildren. Since he fell off a ladder in 2008 he has suffered from pain and needs constant treatment. Murat described his difficult situation: his health insurance provider complains when he takes long periods of sick leave and the employment office will not accept his long absences either. If he gets job offers, however, they come via temporary employment agencies that provide little security. At the time I met him, he was unemployed and struggled to get by with the little money he received:

The cost of living has been rising  … it is so difficult! Where will I get the money we need to pay the rent, to pay the electricity and gas bills? If I don’t pay the bills, they will turn off our electricity and our gas!

Like Murat, many of the people I talked to were in poor health and were in need of frequent appointments with the doctor or hospital visits. Still, the great majority of them were physically capable of performing daily activities without assistance. Yet, two of my female informants had to provide substantial care to their husbands.

The majority lived in privately rented flats that they had moved into several years or even decades ago. Due to ever-rising rents they were not able to move somewhere more suitable as they entered old age. The buildings they lived in were often unmodernised and run-down. One significant problem they faced, particularly those who lived on upper floors, was the lack of elevators in their buildings. Although some of my interlocutors reported close relations with their Austrian neighbours, based on mutual support, often relations were described as rather superficial (for example restricted to greeting each other in the hallway) or, in some cases, even as negative (for example, that Austrian neighbours constantly complained about noise, smell, etc.). They often contrasted these loose relations among neighbours in Vienna to the close relations among neighbours in their Turkish hometowns. I was, however, also told stories of close friendships and mutual support with neighbours who were ready to help and open to friendship.

In the course of my study I visited 12 cultural, political and religious associations (including mosques that also acted as cultural centres) where I conducted participant observation and recorded some of the interviews. While this paper shows how greatly my informants valued the community around these associations, which were central to their sense of social embeddedness, it does not claim that all older Turkish labour migrants and their spouses are socially well-embedded. Two of my interviewees were not able to visit any of the associations on a regular basis because they had to look after a sick or handicapped adult child. Furthermore, the location of this study is of relevance: Vienna, with its historical particularities including its long history of immigration, marks itself off from other places in Austria in terms of opportunity structure. The same can be said with regard to access to migrant associations, which is easier in Vienna than in some other places in the country. These territorial inequalities and different opportunity structures across a national territory need to be considered (Caglar 2013).

The associations I visited differed greatly from one another. However, a common factor was that they were places with a strong sense of community and plenty of room for socialising and different activities (e.g. dancing and music classes). Among them were Turkish cultural centres often also used as mosques, an Islamic-Alevi association^6^ and a Turkish democratic labour association. The last two were the most politically active among the associations I visited (for example, they were used for organising demonstrations and discussion rounds). In these voluntary associations, members aged 50 years and older took on a central role in community building. During weekdays, the great majority of visitors were pensioners (particularly male pensioners) but during weekends and for religious festivities the associations hosted people of all ages and entire families. Regardless of the association people were members of, they most often ascribed the association a key position in their life, as I will show below. Due to the many different associations I include in my analysis here, it is not possible to discuss the specific histories, outlooks and political aspirations of each.^7^ It would also go beyond the scope of this paper to discuss the meaning of voluntary associations for migrants more generally (see Moya 2005). The focus thus remains on the meaning of such associations for older migrants and their experiences of social embeddedness.

## Ageing and social embeddedness

The everyday lives of my informants consisted of daily routines as well as of more exceptional but periodic activities, such as religious holidays celebrated with family and repeated commutes between Austria and Turkey. The older generation continued to feel responsible for their children’s well-being long after they had moved out from their parents’ home. They, for example, cooked lunch for their children when they were busy at work and cared for grandchildren. They cared for adult children and grandchildren as well as for spouses. Even if mothers/grandmothers took over a disproportionate share of care activities, fathers/grandfathers involved themselves increasingly in caring for the younger generations after retirement. Ahmet, one of my informants in his mid-sixties, even became a ‘foster father’ for two small children. When the children’s mother, a friend of his daughter, became seriously sick, he volunteered to care for the children almost on a daily basis while his wife was still working. Ahmet mostly referred to his foster children as his little ‘Austrians’ (since their mother is from Austria) and proudly told me that by now they understand Turkish perfectly.

The older generation not only cared for the younger but also received help from them – for example, with bureaucratic procedures and translation, such as at doctor’s appointments. Many spent time with their children (and grandchildren) at least every week and sometimes even every day, for example, for a home-cooked shared lunch. For religious holidays it was particularly important to be together with the family, as expressed by Erkan when I asked him where he wished to live after his imminent retirement:

That is a big question mark  … mostly here [Vienna] not in Turkey. […]. For example next week we are going to Turkey on vacation for two-and-a-half months at the most but then we’ll return again. This is unfortunate  … there [Turkey] is my country, but my life has been here [in Vienna] that’s why. Moreover, my children are here  … in three months we have two big religious holidays, then we have to be with our children.

Besides visiting family members, an integral part of everyday life for most of the older generation I talked to were the visits to a cultural, religious or political association, in which they actively participated. The associations, which they visited on a regular basis, took centre stage in their lives and provided an important place for them to socialise and to experience social embeddedness outside of the family. The atmosphere in these associations was rather informal and people knew each other well. There they socialised but also shared information and helped each other out with advice. Some described the centre as their second home. These associations greatly differed in nature, particularly with regard to their religious and political orientation, and thus people purposely chose one over the other. Even if in the end these associations catered to similar personal needs and desires, particularly among older Turkish labour migrants, it would be wrong to analyse them as ethnic associations (as in the debate about ethnic resources introduced above). This will be illustrated in the remaining text.

For practising Sunnis, the day was structured by the five prayer times. Regardless of whether I visited practising Sunnis at home or in a cultural centre or mosque, our conversations and also interviews were regularly interrupted during prayer times. I was, however, always welcomed to stay in the room with them for the duration of the prayer. Many of my Sunni informants, especially men but also women, visited their cultural centre/mosque in their neighbourhood for joint prayers on a daily basis. This was also the case for Elif, a 61-year-old woman, who told me the following about her everyday life:

I get up in the morning, wash my face and my hands and go to the mosque. I read the Qur’an. I go there at nine, and at twelve I usually leave the mosque again. When I am home I tidy the flat, I pray and after that I prepare food for my children for when they come. So I have to cook, wash the dishes, clean the flat and pray the afternoon and evening prayer, and these are my days. On Friday I meet with friends in one of our flats. Ten or eleven of us meet at home to read the Qur’an.

For Elif, daily visits to the mosque and its associated cultural centre form an essential part of her everyday life. Her mosque is located in a basement and has an informal atmosphere. Although Elif is active in community life and helps organise the big religious festivities celebrated there, she meets her female friends from the mosque/cultural centre in one of their private homes instead. Religion and the studying of religious texts (Qur’an) are central to the relationships Elif maintains with others from the mosque/cultural centre. She also told me that visitors with an origin other than Turkish are welcome to join prayers there, and she stressed that shared religion rather than ethnicity characterises the community.

Facing financial hardship, my informants rarely, if ever, met friends in cafes or went out with family for dinner in a restaurant. Most often my female informants met friends in private homes; however, my male informants were more likely to meet friends in one of the associations, where sometimes they could even enjoy a warm meal together. It was there that they had the freedom to sit with friends, chat or play cards, without any pressure to buy food or drinks. Most of these associations and mosques were rather invisible from the outside, often being located in basements. When the cultural centre also hosted a mosque, there was a big area reserved for praying which was decorated with carpets and pictures. The remaining space was set aside for socialising and meetings; furnished with tables and chairs, it most often included a small bar where tea/soft drinks and snacks could be consumed.

This was also the case in the Turkish Islamic cultural association, located in a traditional working-class district that 64-year-old Hasan visits on a daily basis. This centre has become a second home to him and he spends most of his day there. Hasan comes to the centre every day to socialise and to join shared prayers. When asked to describe a typical day, Hasan answered:

When I wake up in the morning I pray and then go to bed again, two hours. Afterwards I come here. I stay here until the evening prayer, and then I go home.

Hasan’s children at the time of the interview studied in Turkey and his wife had moved to Turkey to support them. Hasan visits them regularly and each time stays for several weeks before returning to Austria. For 10 years Hasan has been receiving an invalidity pension, which at the time we spoke was 760 Euros a month.^8^ Hasan told me how difficult it is to live on such a small pension. The Turkish Islamic cultural association provides him with a place where he can spend his days, socialising and engaging in the association’s activities regardless of his limited budget. He can even enjoy a warm lunch there for only a small financial contribution. There Hasan meets other men of similar age and with a similar migration history – men who came to Vienna during young adulthood as labour migrants and who have now entered retirement. On warm days Hasan meets his friends from the Turkish cultural association at different public places in the neighbourhood, in a particular park or on a particular park bench. He said he knows where to find them without agreeing on a date and time to meet in advance.

While Hasan, first and foremost, goes to the association to socialise, many of my informants take an active part in the organisation of the association, as is, for example, the case with Mehmet, who is a political and active member in one of the Islamic-Alevi associations. The following quotes from an interview I conducted with Mehmet describe how, besides family and household duties, active participation in community life takes centre stage in his everyday life:

Well, before retirement we had time but now I am a pensioner and I do not have time anymore [laughs]. I have built a house with a garden and a garage; I just planted five or six fruit and vegetable plants. I work in the garden and mow the lawn. When I find time during the week I come here to the association [Islamic-Alevi association]. I have to come here at least twice a week. I help out here as much as I can.

When Mehmet, who himself had not had the chance to learn a profession and worked as unskilled labour in Vienna, talks about his children, their educational and professional success is central. All of them have completed higher education. Besides his family, his Alevi faith and the Alevi association play an important part in Mehmet’s life. In the association he is involved in the organisation of various cultural and political activities:

I come here to the Alevi association regularly … sometimes we have festivities. Sometimes, for example recently, we organised a picnic. It was an Alevi celebration, which was also broadcast on TV. I helped organise it. And next week we have a theatre performance, which I am also organising. Next week on Thursday there will be a demonstration, which we [from the Alevi association] also co-organise. It is a protest against Erdogan and we have to support it!

Mehmet is convinced that his decision to come to Austria was the right one, particularly because as an Alevi he feels discriminated against in Turkey. Mehmet clearly positions himself as being in favour of democratic rights, rights he said he is not granted in his country of origin. For Mehmet, who does no longer call Turkey his home, the Alevi association has become a crucial part of his identity. The Alevi association is not only a religious place and somewhere to socialise but also one where he can get actively involved in political activities that are most often directed towards politics in Turkey, as described above. By expressing his wish to be buried in Vienna (and not in Turkey as most of the Turkish older migrants I talked to preferred), he once more underlined how much he distances himself from present-day Turkey. Almost all of my other informants wished to be buried in their Turkish hometown. For this reason many pay a certain amount to a burial fund (through the association) on a monthly basis, so that their funeral in Turkey would be organised.

Mehmet’s Alevi identity is closely tied to his political ideals and his democratic values. These shared beliefs, he said, are crucial in the Alevi community.

We come here and talk like friends. All of the people here are like brothers and sisters to me … of course it could happen that someone here might have bad intentions, that someone might say something bad or make a mistake. The only one who doesn’t make mistakes is Allah. Everyone makes mistakes … But we come here, first and foremost, to get together, to play music and take part in plays. We cook together, enjoy ourselves. Here we get into a good mood.

For those who lose their spouse in old age, a cultural, political or religious association is likely to play an even more important role. This seems to be the case for 54-year-old Ada, who was widowed 10 years previously. Ada, a self-declared atheist, described the community around the Turkish democratic labour association she is an active member of as extremely important for her well-being and sense of social embeddedness. Ada was forced into invalidity retirement due to bad health. Before she retired, she worked for different cleaning companies and in the home care service sector. Since her husband died, Ada has experienced great loneliness and financial hardship. The only place where she finds some respite is at the political centre of which she is an active member. As she told me:

I had to claim an invalidity pension due to my illness. I would have preferred to work longer because when you work you also socialise. Now, the only thing I have is the association; there, at least, I have something to do. Otherwise I would only sit at home alone or visit my sisters. But I also have financial difficulties, great financial difficulties!  … I have nothing left to hold on to since my husband died. But at least there [in the association] I feel good. When I am at the centre, I am relieved and find distractions. There I can forget everything and put everything aside.

Even though Ada is only in her mid-fifties, she has been an active member of her association for decades already. She, so to speak, grew old together with the centre and with the other founding members. While the association has always been an important place for Ada, its importance increased drastically after her early retirement and the death of her husband. Although Ada’s feelings of loneliness set her apart from the other informants, she described the regular visits to the association she is a member of as the highlights of her week, as did many of my other informants in regard to their associations. The strong social ties and the support network among their members was stressed by my interviewees in statements such as ‘We always ask each other for advice when something comes up’ or ‘When something happens, all of us are there to help!’ Even if there is a strong network and people help each other out as well as they can, the community is only accessible as long as people are mobile enough to come on a regular basis to one of the associations. In the following section, I will thus explore perspectives on old age and late life care.

## Transnational outlooks on age: dual orientation

As mentioned in the introduction, the period around retirement was a crucial stage of life for renegotiating transnational living arrangements. Personal preferences were balanced with family needs and desires and most opted to remain in Austria while spending extended periods of the year in Turkey. While the great majority of my informants’ children and grandchildren lived in Austria, their parents (if still alive) in most cases remained in Turkey. My informants often felt uncomfortable about not being with their parents, especially when the latter were in need of care. Sometimes parents were invited for an extended stay in Austria with their children and grandchildren. My informants supported their parents in Turkey through gifts and remittances. The ways of staying in contact with relatives over great geographical distances varied and benefited from new technologies including mobile phones and text messaging, and in some cases also Skype (see Baldassar 2007; Baldock 2003; Vertovec 2009). In addition to this regular communication, relatives in Turkey were the subject of conversations, not only within the family but also at the association where news about relatives in Turkey was frequently exchanged. In this way relatives not physically present in Vienna were nevertheless present in thoughts and everyday conversations. My informants also remained connected with their country of origin through satellite TV, which enabled them to watch numerous Turkish TV channels. This dual orientation or ‘bifocality’ characterises everyday experiences of transnational living (Vertovec 2004).

This dual orientation was also present when articulating ideas and preferences for late life care, which was located between care carried out by family members and more institutionalised forms of care in Austria (as well as Turkey). While there certainly was a strong support network among members of the associations I visited, these networks seemed only accessible to my informants as long as they were mobile enough to join the community by visiting the association regularly. When I asked my interlocutors where and from whom they would seek help if and when in need of care, they hardly ever mentioned the community around one of the associations. Almost everyone hoped that their children would take care of them when in need. Many, however, were aware that they could not take their children’s help for granted and knew that ‘times have changed’. They knew that the younger generation was busy with jobs (or with seeking a job) and with raising their children and had limited time left to care for older family members. Most of the people I talked to added that they would seek institutional care if they needed to. But they had no information about how to secure a place in a nursing home nor did they have a clear idea of how these homes were run. In the end, they were sceptical about whether a nursing home in Austria would satisfy their needs, as expressed by 53-year-old Hülya:

I don’t know; only Allah knows. We indeed hope that our children can take care of us. […] But if my children don't take care of me I can’t force them to. […] We have not visited a nursing home yet nor has anyone from among our family or friends. Therefore we don’t know anything about them – neither good things nor bad. […] We would need to know whether we could practise our religion freely there. They are Christians; I am Muslim. I don’t know whether they would give me a place to pray there … or if they would give me a place to read the Qur’an; nobody knows.

Besides questions of faith (e.g. prayer rooms), my interlocutors raised language difficulties as an issue when considering a nursing home in Vienna. Also halal food was an important topic. Scepticism towards Austrian nursing homes was common not only among Muslim informants but also among those describing themselves as atheists.

## Discussion

As has been shown in this paper, political, religious and cultural associations are crucial to many older migrants and their sense of social embeddedness. Migrants who have entered retirement age and whose children have moved out from their parental home are key actors in community building around these associations. This has not yet been sufficiently acknowledged in the relevant literature. Moreover, these associations are not only relatively easy to access in old age but also provide a social context for the integration of personal histories and transnational life concepts (see Palmberger and Tos˘ić 2016). The importance of such migrant associations, however, needs to be further explored in connection to age and the life course in different (regional) contexts (see also Ciobanu and Fokkema and Patzelt in this issue).

Older labour migrants, who were not or were no longer integrated in the job market and whose children had moved out, found in these associations a place to socialise with others of their generation who shared a similar migration history and interests. Rather than withdrawing from society as suggested by disengagement and activity theories (see Fennell, Phillipson, and Evers 1988; Jamieson 2002), my informants actively engaged in community life and undertook important voluntary work in one of the associations as well as supported their children and grandchildren. This does not mean, however, that isolation among older migrants does not exist. My informants were still mobile and able to visit one of the associations. It is likely that migrants (like non-migrants) experience isolation more often in advanced old age once they become less mobile (see Fokkema and Naderi 2013). This also corresponds with my informants’ own ambiguity about their future, especially in case of restricted mobility and their concerns about the lack of adequate institutional care.

Despite the positive influence of voluntary associations, I have shown, along with other authors (see, e.g. Jamieson 2002; Warnes, Warren, and Nolan 2000; White 2006), how older labour migrants face precarious living conditions and are exposed to several vulnerabilities, due to their age but also due to their socio-economic status (e.g. precarious jobs, low income/pension and flats in substandard buildings) as well as their migration background (e.g. lack of language skills and minority religion) and gender. My informants stressed in particular the lack of suitable (culturally sensitive) care in old age as one uncertainty older Turkish labour migrants face. As shown in this paper, there is an increasing awareness that children may not be able to look after their parents when they need care. Moreover, some of the older migrants also critically assessed that family care is not necessarily better than that provided by a professional carer, and the desire for suitable institutional alternatives was expressed.

It is generally assumed that migrants’ ‘ethnic’ compensation strategies lie in the well-functioning support network of the extended family (as opposed to the so-called ever-weakening family support structures of non-migrants in Western Europe), particularly in late life. As different authors have shown (Baykara-Krumme 2007; Fokkema and Naderi 2013), older people’s family relations differ less between migrants and non-migrants than is often assumed. Just as family support networks among migrants are exaggerated, family support networks among non-migrants are underplayed. Such a picture of family solidarity first and foremost provides policy-makers with a good excuse to delay far-reaching reforms that would eventually reduce institutionalised forms of discrimination, particularly in regard to late life care.

This means it would be wrong to characterise strong family relations among older migrants as an ‘ethnic resource’. Similarly, it would be problematic to speak of the voluntary associations as an ethnic resource. As I have shown, the community around the voluntary associations was not first and foremost defined by ethnic affiliation. In fact, political and religious affiliations were most decisive for the choice of a particular association, although Turkish language dominated and thus was more or less a prerequisite for becoming part of the community (only in mosques, which non-Turkish visitors sometimes attended, did language play a somewhat lesser role). The different associations reflect the diversity among older Turkish labour migrants, suggesting we take seriously ‘migrant’s agency beyond ethnic subjects’ (Caglar 2013, 407).

To summarise, the social ties people maintain in the associations described are not particularly ‘ethnic’ but rather are based on shared religious and political beliefs as well as a common generational identity, defined by similar migration histories, socio-economic location and shared ideas of transnational ageing. In this paper, I have shown how older labour migrants are key figures in Turkish cultural, religious and political associations, which are again essential for strengthening social ties and for their sense of social embeddedness. Thus, it can be said that a shared stage of life together with a shared migration history, transnational living arrangements, similar religious, cultural and/or political ideas (varying between the different centres) and a common mother tongue strengthen a sense of social embeddedness for older migrants who are members of a particular voluntary association.

## Notes

1. While according to the census of 2001, already 25% of Vienna’s population of 50 years of age and older were first-generation migrants (approximately half of them migrated from former Yugoslavia or Turkey, mostly as so-called guest workers), demographic forecasts predict that this trend will continue and that in 2021 around half of the population of over-fifties will have been born in a country other than Austria (Reinprecht 2006, 12).

2. Exceptions include, for example, Alber and Drotbohm (2015), Bolzman et al. (2004), Burholt et al. (2000), Gardner (2002), Hromadzic and Palmberger (Forthcoming), Oliver (2004), Sokolovsky (1985), Torres and Karl (2016) and Walsh and Näre (2016).

3. My female informants generally had a lower level of education than my male informants. Many of the women interviewed had only completed a couple of years of primary school, some had no school education whatsoever.

4. The ages given in this paper are the actual ages and not the age stated on their birth certificates. The latter in many cases differs from the actual date of birth by up to several years due to late registration of the child’s birth or other reasons.

5. In order to protect my informants’ anonymity, the names of all the people introduced in this paper were changed as well as places and other personal details whenever necessary.

6. Since 2013, Alevis have been a recognised religious group in Austria.

7. Several scholars have critically assessed the historical and political dimensions of migrant associations. Caglar (2006), for example, analyses how hometown associations have their specific histories and political locations. Yurdakul (2009) analyses in detail different political engagements upheld by different immigrant associations in Germany and stresses that they develop their own political agendas and decisions rather than being passive recipients of state politics.

8. A disproportionately high share of those receiving invalidity pensions in Austria are migrants (Reinprecht 1999).
